# Continuous glucose monitoring reveals high prevalence of hyperglycaemia in patients prior to pancreatic surgery: A pilot study

**DOI:** 10.1016/j.jcte.2025.100426

**Published:** 2025-12-03

**Authors:** Heleen Driessens, Josephine A.C. Woldring, Maarten W. Nijkamp, Peter R. van Dijk, Joost M. Klaase

**Affiliations:** a Department of Surgery, Division of Hepato-Pancreato-Biliary Surgery and Liver Transplantation, University Medical Centre Groningen, Groningen, the Netherlands; b University of Groningen, Groningen, the Netherlands; c Department of Internal Medicine, Isala, Zwolle, the Netherlands; d Department of Internal Medicine, University Medical Centre Groningen, Groningen, the Netherlands

**Keywords:** Diabetes mellitus, Pancreatic cancer, Postoperative complications, Prehabilitation

## Abstract

**Aims:**

Patients with pancreatic tumours are at increased risk of diabetes mellitus (DM) and hyperglycaemia and a subsequent higher risk of developing postoperative complications. Continuous glucose monitoring (CGM) can be used to assess the prevalence of hyperglycaemia in pancreatic surgery patients.

**Methods:**

This single-centre observational pilot study (2023–2025) included 15 patients with DM type 2 or new onset DM (HbA1c ≥ 48 mmol/mol (6.5 %)) undergoing pancreatic surgery. Blinded CGM was performed for 2 weeks preoperatively and 2 weeks perioperatively. Primary outcome was time above range (TAR)(glucose > 10.0 mmol/l (180.2 mg/dl)) as a percentage of total CGM wear time. Secondary outcomes were time below and in range, glucose metrics, difference in preoperative HbA1c and patient satisfaction regarding CGM wear.

**Results:**

In total, 5 patients had new-onset DM, 6 suboptimal controlled DM (HbA1c ≥ 53 mmol/mol (7.0 %)) and 4 optimal controlled DM (HbA1c < 53 mmol/mol (7.0 %)) at baseline. Median preoperative TAR (>10.0 mmol/L (180.2 mg/dl)) was highest in the suboptimal controlled DM group (59.7 % [35.1–68.6]), compared to 7.9 % [0.9–19.4] in the optimal controlled and 16.7 % [7.7–23.7] in the new-onset DM group. Perioperatively, the optimal controlled DM group had the highest TAR (26.7 % [11.3–49.0]) while the new-onset and suboptimal controlled DM group had TARs of 4.6 % [1.2–9.6] and 16.3 % [11.5–23.4], respectively.

**Conclusions:**

Blinded CGM revealed frequent pre- and perioperative hyperglycaemia and high inter-individual variability in TAR among patients with (new-onset) DM undergoing pancreatic surgery. These findings support the need for stricter and more individualized glucose monitoring. This could optimize preoperative glucose management and thereby possibly reduce postoperative complications.

## Highlights

• Blinded CGM revealed frequent hyperglycaemia in pancreatic surgery patients.
• Substantial inter-individual glycaemic variability was observed among patients.
• Hyperglycaemia occurred even in patients with optimal controlled diabetes.
• CGM was feasible and well tolerated by pancreatic surgery patients.
• CGM may help improve perioperative glucose control and reduce complications.

## Introduction

Perioperative hyperglycaemia is associated with adverse surgical outcomes, especially infections, impaired wound healing, but also delayed gastric emptying (DGE) [[1], [2], [3], [4], [5], [6], [7]]. Yet, the prevalence of hyperglycaemia in surgical patients ranges from 20 % to 35 % and is also present in persons without a previous history of DM [2,8]. In patients with pancreatic cancer, the prevalence of hyperglycaemia can be even as high as 40 %, often with recent onset as a result of tumour-derived influence on the glucose metabolism [9,10]. For these patients, surgical resection remains the only curative option, but pancreatic surgery is associated with major postoperative complication rates up to 33 % [11].

Recent studies have focused on preoperative interventions to optimize modifiable risk factors in order to prevent postoperative complications, known as prehabilitation [[12], [13], [14], [15], [16]]. These prehabilitation programs typically address physical fitness, nutritional status, psychological resilience, anaemia, and intoxications. However, hyperglycaemia is often overlooked in these prehabilitation efforts. Since patients with pancreatic cancer with perioperative hyperglycaemia are at an even higher risk of developing postoperative complications, preventing perioperative hyperglycaemia may reduce postoperative complications in this patient population [17].

In order to prevent hyperglycaemia, it is important to be able to monitor blood glucose levels to identify (imminent) hyperglycaemia. To date point-of-care (POC) capillary blood glucose measurements (BGM) are often used for this purpose. BGM has been shown to be able to identify hyperglycaemia in the perioperative period and that hyperglycaemia was independently associated with postoperative complications and mortality [3,4,7,18]. However, BGM may fail to detect blood glucose fluctuations, missing hypo- and hyperglycaemic episodes. Another measure, glycated haemoglobin (HbA1c), assesses long-term glycaemic control over three to four months. A previous study has shown that preoperative elevated HbA1c was associated with postoperative complications following major abdominal surgery [19]. Nevertheless, HbA1c has its limitations with respect to identifying glucose excursions and modest hyperglycaemia and might therefore be less effective for monitoring perioperative blood glucose levels.

Given the limitations of BGM and HbA1c in perioperative glucose control, continuous glucose monitoring (CGM) is emerging as a promising alternative to monitor glucose levels. Continuous glucose monitoring (CGM) devices provide real-time, continuous glucose measurements via subcutaneous sensors that assess interstitial fluid glucose [20]. In outpatient diabetes care, CGM has already demonstrated significant benefits, including improved glycaemic control and reduced hypoglycaemia, improved quality of life, increased treatment satisfaction and less diabetes related admissions [[21], [22], [23]]. In hospital settings, CGM has increasingly been recognized as a valuable tool by offering better (i.e. earlier and more detailed) detection of glucose fluctuations, enabling timely interventions to maintain optimal glucose levels [24,25].

Currently, no evidence exists on pre- and perioperative glucose levels measured by CGM in pancreatic cancer patients. We hypothesize that CGM can identify hyperglycaemia in surgically treated patients with pancreatic tumours with either new-onset or pre-existing diabetes, even in those without seemingly high risk of hyperglycaemia based on their HbA1c levels. This study will provide insights into the prevalence and duration of hyperglycaemia in this patient population.

## Subjects, materials and methods

### Study design and setting

This prospective observational pilot study was conducted at the University Medical Centre Groningen (UMCG), Groningen, the Netherlands from June 2023 until January 2025. The study was approved by the Regional Ethical Committee (identifier 15819). All patients gave written informed consent before inclusion.

### Participants

Patients scheduled for elective pancreatic surgery (pancreatoduodenectomy, distal pancreatectomy or total pancreatectomy) for periampullary (pre)malignant tumours with pre-existent diabetes mellitus DM type 2 or new-onset DM were recruited at the preoperative outpatient clinic. Patients were considered having new-onset DM when HbA1c was ≥48 mmol/mol (6.5 %), without a prior diabetes diagnosis documented in the patient’s medical record or use of glucose-lowering medications [26]. Although previously undiagnosed or undertreated diabetes cannot be fully excluded, this definition aligns with current guidelines [26]. An elevated level of HbA1c for patients with pre-existent DM type 2 was defined as HbA1c ≥53 mmol/mol (7.0 %) [27]. Based on diabetes status and HbA1c level, patients were divided into 3 groups: 1) new-onset DM, 2) pre-existent DM and elevated HbA1c (hereafter referred to as ‘suboptimal controlled DM’ group) and 3) pre-existent DM and normal HbA1c (hereafter referred to as ‘optimal controlled DM’ group).

### In- and exclusion criteria

Inclusion criteria were age ≥ 18 years, (suspicion of) a pancreatic malignant or premalignant tumour requiring pancreatic surgery, scheduled for elective pancreatic surgery for their periampullary tumour at the UMCG, having a history of type 2 DM or new-onset DM and having a life expectancy of more than 6 months. Exclusion criteria were acute surgery (within 2 weeks after initial visit to their surgeon), type 1 DM, steroid induced or monogenetic forms of diabetes, receiving neoadjuvant therapy during the intended study period, undergoing surgery in another hospital, insufficient understanding of the Dutch language and known allergy to acrylates (substance in the CGM attachment material).

### Prehabilitation program

Within the UMCG at the outpatient clinic of the department of HPB surgery, a multimodal prehabilitation program is standard of care in the preoperative care pathway. All patients scheduled to undergo HPB surgery were referred to our outpatient prehabilitation clinic and assessed on six patient-related modifiable risk factors: (1) low aerobic capacity, (2) malnutrition, (3) low psychological resilience, (4) iron deficiency (anaemia) and hyperglycaemia, (5) frailty, and (6) substance abuse (i.e., tobacco use and/or alcohol consumption) [28]. Based on the screening and assessment, patients received an intervention for each risk factor that was present. The full details on the screening, assessment and interventions have been described previously [[28], [29], [30]]. All patients in this study participated in the prehabilitation program and were assessed for the presence of DM and hyperglycaemia. If persons had a history of DM or hyperglycaemia (defined as a HbA1c ≥ 53 mmol/mol (7.0 %) in persons with a history of DM or HbA1c ≥ 48 mmol/mol (6.5 %) in persons without a history of DM) was present, they were referred to the outpatient diabetes clinic. At the clinic, a specialized nurse educated all patients about DM, the effect of elevated glucose levels on postoperative outcomes and the risk of developing new-onset DM after pancreatic surgery. Furthermore, patients received education on how to use their current medication or how to better regulate their glucose levels with lifestyle advice. If necessary, glucose lowering medications were prescribed or current medications were adjusted.

### Study procedures

**Fig. 1:**
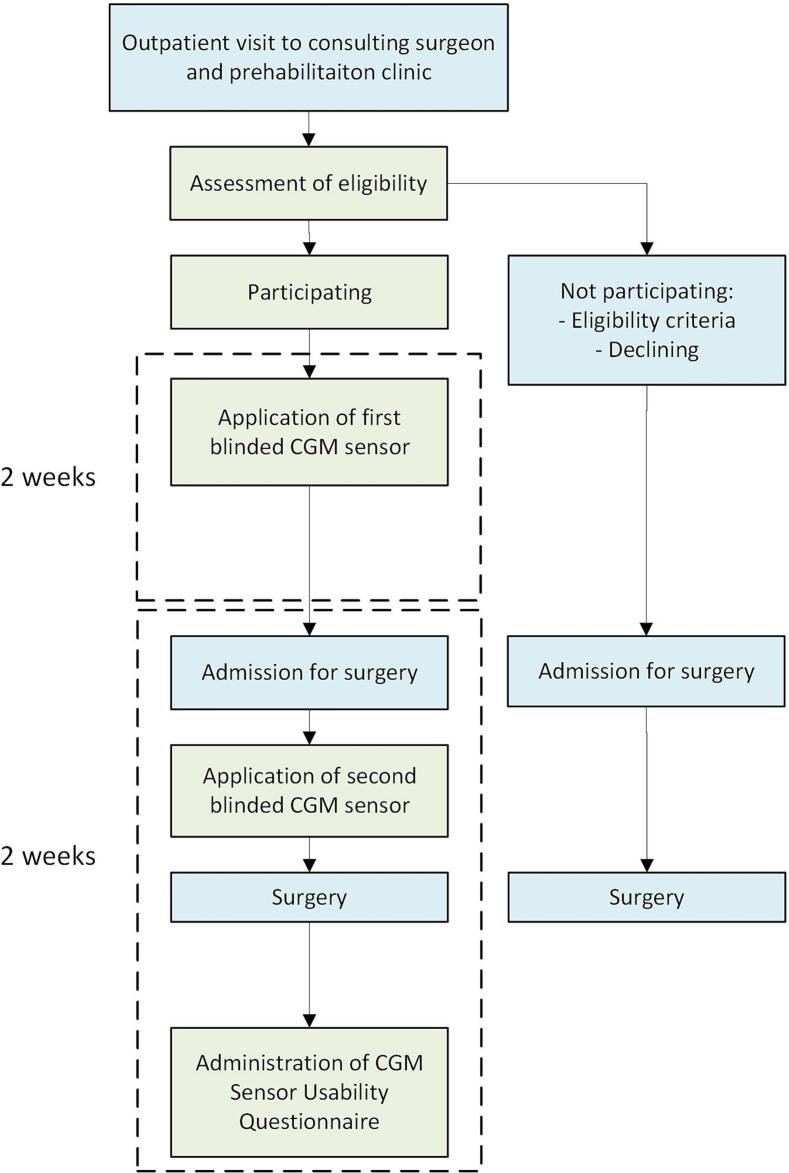
Study timeline. CGM = Continuous Glucose Monitoring.

During the study period, a blinded CGM sensor was applied to the participants for 2 weeks preoperatively and 2 weeks perioperatively (starting from one day before surgery until 2 weeks postoperatively). A study timeline is presented in Fig. 1. For CGM, the Freestyle Libre Pro system (Abbott Diabetes Care Ltd., Witney, Oxon, UK) was used, which is placed into the subcutaneous tissue and measures glucose levels from the interstitial fluid. This sensor measured glucose levels every 15 min, for 24 h a day during a 14-day period. Measurement outcomes were blinded for the patient and healthcare personnel and were not used for treatment purposes. The sensor data was obtained when the final period of CGM was completed. Once patients consented to participate in this study, a study visit was planned to apply the first CGM sensor to the upper arm of the patient. At the day of admission for surgery, the second sensor was applied. After removal of the sensors, data was obtained by the Freestyle Libre Pro reader and exported via Libreview software, version 3.18 (Abbott Diabetes Care Ltd., Witney, Oxon, UK).

To assess patients’ satisfaction with blinded CGM sensor wear, the CGM sensor usability questionnaire was administered to all participants via the electronic data management system REDCap following completion of the final CGM period. The questionnaire was adapted from Massa et al. [31], whereby the final five questions from the original questionnaire were removed, as these were not applicable to our study population since the outcomes of the CGM sensor were blinded. Participants rated their agreement with 10 statements regarding the CGM sensor using a 5-point Likert scale ranging from 1 (strongly agree) to 5 (strongly disagree). The adjusted questionnaire can be found in Supplementary File S1.

### Perioperative management of blood glucose levels

As part of standard care, all patients with DM were admitted the day before surgery. A specialized diabetes nurse reviewed medication and all glucose lowering medications were paused on the day of surgery. Intraoperatively, glucose levels were monitored hourly, with insulin administered as needed based on local protocol aiming at glucose levels between 5 and 10 mmol/l (90.1 and 180.2 mg/dl). Postoperatively, glucose was monitored 4-times daily using POC BGM. Glucose lowering medications were continued when oral intake was resumed. A specialized nurse was continuously involved in glucose management, with adjustments to glucose lowering therapy made as necessary, aiming at glucose levels between 5 and 10 mmol/l (90.1 and 180.2 mg/dl) using correctional insulin administration.

### Perioperative nutrition

All patients were given instructions to maintain a fasting period of a minimum of six hours for food and a minimum of two hours for clear liquids prior to the scheduled surgery according to standard care. After surgery, patients resumed oral intake as soon as possible in case of a distal pancreatectomy or total pancreatectomy. In case of a pancreatoduodenectomy, patients received only 500 mL of water per nasojejunal tube for the first 24 h, after which gradually tube feeding was introduced. As soon as the nasogastric tube could be removed, oral intake was gradually increased and tube feeding reduced.

### Outcomes

The primary outcome was sensor-derived time above range (TAR)(sensor measured glucose > 10.0 mmol/L (180.2 mg/dl)) in percentage of total CGM-wearing time during the preoperative and perioperative period. For this study, the perioperative period was defined as the interval from 1 day before surgery up until 13 days after surgery. Secondary outcomes were time within range (TIR)(glucose 3.9–10.0 mmol/L (70.3-180.2 mg/dl)), time below range (TBR)(glucose < 3.9 mmol/L (70.3 mg/dl)), and time above > 13.9 mmol/L (250.5 mg/dl) in percentage of time, duration of episodes (an episode was defined as ≥ 15 min below or above threshold) of TBR, TAR and time above > 13.9 mmol/L (250.5 mg/dl), difference in baseline and perioperative (measured on day of hospital admission for surgery) HbA1c, mean sensor glucose levels, coefficient of variation (CV) and standard deviation (SD) of glucose during the preoperative and perioperative period. Patients’ satisfaction measured with the CGM sensor usability questionnaire was also assessed.

### Other study variables

Next to study outcomes described above, baseline patient characteristics, glucose lowering medication use, history on DM, comorbidities, ASA-classification [32] and Charlson Comorbidity Index (CCI) [33] were collected. Perioperative variables included type of surgery, open or minimally invasive surgery, duration of surgery and blood loss during surgery. Postoperative outcomes included all complications (graded by the Clavien-Dindo classification, whereby a major complication was defined as Clavien-Dindo grade ≥ 3a) [34], length of hospital stay in days, readmissions within 30 days after discharge and mortality within 30 days after discharge. Adverse events related to CGM wear were also collected.

### Statistical analysis

Sensor data cleaning and derivation of glucose metrics were performed using R (version 4.4.1, R Foundation for Statistical Computing, Vienna, Austria) with the cgmanalysis package version 3.0. [35]. Data was analysed and visualized using GraphPad Prism for Windows (version 9.1.0, GraphPad Software, San Diego, CA, USA). Data was presented in tables and figures. Nonparametric data was presented with median and interquartile range (IQR) or mean and standard deviation (SD), as appropriate. Categorical data is summarized by frequency and percentage. Descriptive statistics were used to present data on the primary and secondary outcomes per DM group and for the total study population. Descriptive statistics were used to present data on patient characteristics, perioperative and postoperative variables.

## Results

### Baseline characteristics

A total of 15 patients were included: 5 with new-onset DM, 6 with suboptimal controlled DM and 4 with optimal controlled DM. The median age was 71.0 years [62.0–75.0], 8 patients (53.3 %) were female, and the median BMI was 27.4 kg/m^2^ [24.3–28.9]. The median CCI was 4.0 [3.0–5.0]. All tumours originated in the pancreas, with the majority being adenocarcinomas (73.3 %). Six patients received neoadjuvant chemotherapy and were included after completion of neoadjuvant chemotherapy. Of the 15 patients, 10 were referred for a preoperative diabetes outpatient consultation. In 5 of these patients, glucose lowering medication was adjusted, ranging from start of short- and long-acting insulin to adjusting the dosage of oral glucose lowering medication. The other 5 patients received counselling on postoperative glucose regulation and long-term diabetes management only. All baseline characteristics can be found in Table 1.

**Table 1:** Baseline characteristics for all patients and by DM group.

	All patients (N = 15)	New-onset DM (n = 5)	Suboptimal controlled DM (n = 6)	Optimal controlled DM (n = 4)
Age, years	71.0 [62.0–75.0]	73.0 [71.0–76.0]	73.0 [54.0–76.0]	62.0 [56.5–67.0]
Gender, female	8 (53.3 %)	3 (60.0 %)	4 (66.7 %)	1 (25.0 %)
BMI, kg/m^2^	27.4 [24.3–28.9]	22.2 [20.8–27.6]	27.4 [24.7–28.6]	28.3 [26.1–29.5]
CCI	4.0 [3.0–5.0]	4.0 [3.0–5.0]	5.0 [4.0–7.0]	3.5 [2.0–4.5]
ASA-score*				
I	0	0	0	0
II	7 (50.0 %)	1 (20.0 %)	2 (40.0 %)	4 (100 %)
III	7 (50.0 %)	4 (80.0 %)	3 (60.0 %)	0
Alcohol use	5 (33.3 %)	2 (40 %)	2 (33.3 %)	1 (25.0 %)
Smoking	2 (13.3 %)	0	1 (16.7 %)	1 (25.0 %)
Histology of tumor				
Adenocarcinoma	11 (73.3 %)	3 (60.0 %)	5 (83.3 %)	3 (75.0 %)
NET	2 (13.3 %)	1 (20.0 %)	0	1 (25.0 %)
MCN	1 (6.7 %)	0	1 (16.7 %)	0
Other	1 (6.7 %)	1 (20.0 %)	0	0
Neoadjuvant therapy	6 (40.0 %)	1 (20.0 %)	2 (33.3 %)	3 (75.0 %)
Baseline HbA1c, mmol/mol	48.5 mmol/mol [35.5–53.0]	51.0 [51.0–61.0]	61.5 [59.0–62.0]	44.0 [39.5–50.0]
Diabetes duration, years	0.5 [0.0–9.0]	0 [0–0]	8.0 [2.0–9.0]	8.0 [0.5–16.5]
Medication				
Lifestyle/diet	3 (20.0 %)	1 (20.0 %)	1 (16.7 %)	1 (25.0 %)
Metformin	6 (40.0 %)	0	3 (50.0 %)	3 (75.0 %)
SGLT-2 inhibitor	1 (6.7 %)	0	1 (16.7 %)	0
GLP1-receptoragonist	1 (6.7 %)	0	1 (16.7 %)	0
Sulfonylurea	1 (6.7 %)	0	1 (16.7 %)	0
Short-acting insulin	5 (33.3 %)	1 (20.0 %)	3 (50.0 %)	1 (25.0 %)
Long-acting insulin	6 (40.0 %)	2 (40.0 %)	4 (66.7 %)	1 (25.0 %)
No treatment	4 (26.7 %)	3 (60.0 %)	0	0
Diabetes complications				
Microvascular	0	0	0	0
Macrovascular	2 (13.3 %)	0	2 (33.3 %)	0

Data are depicted as median [IQR] or numbers (%).

ASA = American Society of Anesthesiologists; BMI = body mass index; CCI = Charlson Comorbidity Index; DM = diabetes mellitus; GLP1 = glucagon-like peptide 1; HbA1c = glycated haemoglobin; IPMN = intrapapillary mucinous neoplasm; IQR = interquartile range; MCN = mucinous cystic neoplasm; NET = neuroendocrine tumour; SGLT-2 = sodium-glucose cotransporter 2.*: For one patient in the suboptimal DM group, no ASA-score was determined.

### Diabetes characteristics

The median baseline HbA1c for the entire cohort was 55.0 mmol/mol [50.5–61.5] (7.2 % [6.8-7.8]). Patients with optimal controlled DM had a lower median HbA1c (44.0 mmol/mol [39.5–50.0] (6.2 % [5.7-6.7])) compared to patients with suboptimal controlled DM (61.5 mmol/mol [59.0–62.0] (7.8 % [7.5-7.8])) and new-onset DM (51.0 mmol/mol [51.0–61.0] (6.8 % [6.8-7.7])). Different types of glucose lowering medications were used across all groups, of which insulin (short- and/or long-acting) was most often used by patients with suboptimal controlled DM. Detailed diabetes-related characteristics are presented in Table 1.

### Preoperative glucose metrics

All 15 patients completed the preoperative CGM period. The median sensor wear time was 12.9 days [9.5–13.9]. The overall median of the mean sensor glucose was 8.0 mmol/L [7.3–10.5] (144.1 mg/dl [131.5-189.2]), with a median SD of 2.1 mmol/L [1.7–2.7] (37.8 mg/dl [30.6-48.6]) and a median CV of 23.9 % [18.9–27.0]. Mean sensor glucose was highest in the suboptimal controlled DM group (10.5 mmol/L [9.3–11.3] (189.2 mg/dl [167.6-203.6])) compared to the optimal controlled DM group (7.4 mmol/L [6.7–8.3] (133.3 mg/dl [120.7-149.6])) and new-onset DM group (7.7 mmol/L [7.5–8.0] (138.7 mg/dl [135.1-144.1])).

**Fig. 2:**
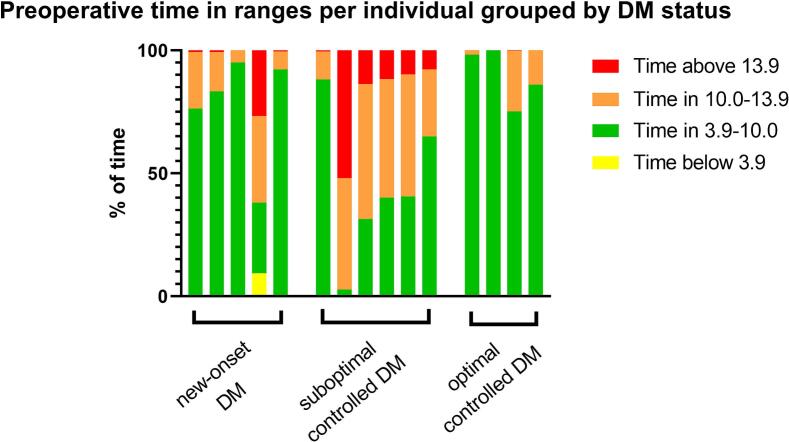
Preoperative time in ranges per individual grouped by DM status. Each bar represents a single patient. Time in ranges are stacked per patient, whereby yellow represents time below range, green represents time in range, orange represents time above range to 13.9 mmol/L and red represents time above 13.9 mmol/L. Time in ranges are presented as % of time. (For interpretation of the references to colour in this figure legend, the reader is referred to the web version of this article.)

The median TIR (3.9–10.0 mmol/L (70.3–180.2 mg/dl)) was 76.0 % [40.3–90.2]. Patients with suboptimal controlled DM had the lowest TIR (40.3 % [31.4–64.6]), whereas those with optimal controlled and new-onset DM had TIRs of 92.1 % [80.6–99.1] and 83.3 % [76.0–92.3], respectively. The median TAR (>10.0 mmol/L (180.2 mg/dl)) was highest in the suboptimal controlled DM group (59.7 % [35.1–68.6]), compared to 7.9 % [0.9–19.4] in the optimal controlled DM group and 16.7 % [7.7–23.7] in the new-onset DM group. TBR (<3.9 mmol/L (70.3 mg/dl)) was observed in only three patients, two with new-onset DM and one with suboptimal controlled DM and all three patients used insulin. All preoperative glucose metrics by DM status are presented in Table 2. Individual time in ranges varied substantially between individuals and are visualized in Fig. 2.

**Table 2:** Preoperative glucose metrics for all patients and by DM group.

	All patients (N = 15)	New-onset DM (n = 5)	Suboptimal controlled DM (n = 6)	Optimal controlled DM (n = 4)
Duration of sensor wear, days	12.9 [9.5–13.9]	13.0 [6.9–13.9]	13.4 [10.8–13.9]	10.7 [9.5–12.9]
Mean sensor glucose, mmol/L	8.0 [7.3–10.5]	7.7 [7.5–8.0]	10.5 [9.3–11.3]	7.4 [6.7–8.3]
SD, mmol/L	2.1 [1.7–2.7]	2.1 [1.7–2.5]	2.6 [2.4–2.7]	1.5 [1.0–1.7]
CV, %	23.9 [18.9–27.0]	26.5 [23.9–30.8]	25.1 [20.9–27.0]	18.5 [14.7–20.9]
Time in ranges, %				
<3.9 mmol/L	0.0 [0.0–0.0]	0.0 [0.0–0.3]	0.0 [0.0–0.0]	0.0 [0.0–0.0]
3.9–10.0 mmol/L	76.0 [40.3–90.2]	83.3 [76.0–92.3]	40.3 [31.4–64.6]	92.1 [80.6–99.1]
>10.0 mmol/L	23.7 [9.8–59.7]	16.7 [7.7–23.7]	59.7 [35.1–68.6]	7.9 [0.9–19.4]
>13.9 mmol/L	0.8 [0.1–10.7]	0.8 [0.5–0.8]	10.7 [7.8–13.8]	0.0 [0.0–0.1]
Frequency of episodes, n				
<3.9 mmol/L				
>10.0 mmol/L	0.0 [0.0–0.0]	0.0 [0.0–1.0]	0.0 [0.0–0.0]	0.0 [0.0–0.0]
>13.9 mmol/L	19.0 [14.5–28.5]	19.0 [18.0–19.0]	24.0 [19.0–31.0]	11.5 [1.0–37.5]
	2.0 [0.0–15.0]	2.0 [1.0–2.0]	19.0 [7.0–24.0]	0.0 [0.0–0.5]

All data are depicted as median [IQR].CV = coefficient of variation; DM = diabetes mellitus; min = minutes; SD = standard deviation.

### Difference in baseline and preoperative HbA1c

**Fig. 3:**
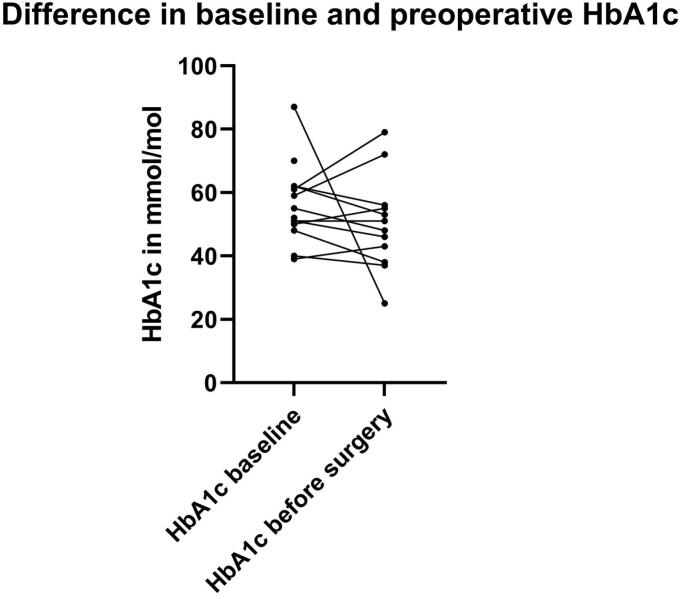
Individual changes in HbA1c at baseline and perioperatively. Each line indicates the change in HbA1c of one individual. For 3 patients, no perioperative HbA1c was available, therefore only 12 lines are shown.

Perioperative HbA1c (measured on day of hospital admission for surgery) was available for 12 patients and had a median value of 49.5 mmol/mol [40.5–55.5] (6.7 % [5.9-7.2]). The median interval between baseline and perioperative HbA1c was 47.0 days [27.0–58.5]. Across all groups, median perioperative HbA1c was lower than median baseline HbA1c, with a median perioperative HbA1c of 48.5 mmol/mol [35.5–53.0] (6.6 % [5.4-7.0]) in the new-onset DM group, 56.0 mmol/mol [53.0–72.0] (7.3 % [7.0-8.7]) in the suboptimal controlled DM group and 38.0 [37.5–40.5] (5.6 % [5.6-5.9]) in the optimal controlled DM group. Individual changes in HbA1c are visualized in Fig. 3.

### Perioperative characteristics

The most frequently performed procedure was a pylorus-resecting pancreatoduodenectomy (PRPD) (53.5 %). In two patients, no resection was performed due to progression of disease. Duration of surgery and blood loss were highest in the optimal controlled DM group. Postoperatively, 5 patients (38.5 %) experienced a major complication, 5 patients (38.5 %) developed a surgical site infection (SSI) and 3 patients (23.1 %) had delayed gastric emptying (DGE). The median length of hospital stay was 8.0 days [6.5–13.5]. All perioperative characteristics by DM status are presented in Supplementary Table S1.

### Perioperative glucose metrics

**Fig. 4:**
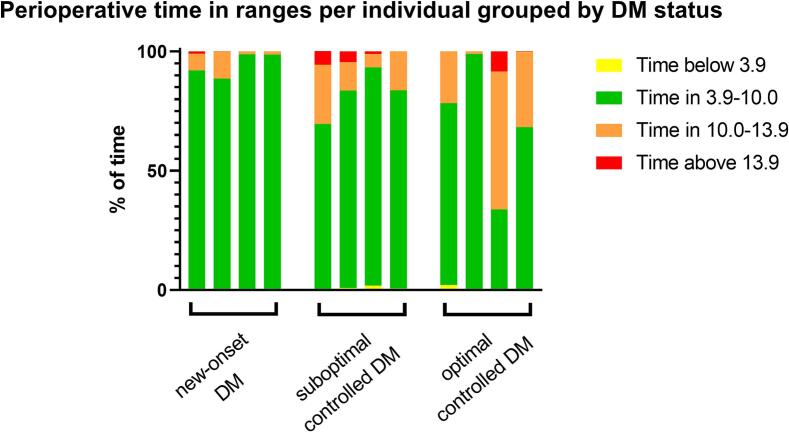
Perioperative time in ranges per individual grouped by DM status. Each bar represents a single patient. Time in ranges are stacked per patient, whereby yellow represents time below range, green represents time in range, orange represents time above range to 13.9 mmol/L and red represents time above 13.9 mmol/L. Time in ranges are presented as % of time. (For interpretation of the references to colour in this figure legend, the reader is referred to the web version of this article.)

Perioperative CGM data were available for 12 of the 13 patients, as one sensor was lost. The median of the mean sensor glucose across all patients was 7.8 mmol/L [6.8–8.7] (140.5 mg/dl [122.5-156.8]). Patients in the optimal controlled DM group had the highest median of the mean sensor glucose (8.3 mmol/L [7.1–9.9] (149.6 mg/dl [127.9-178.4])) compared to the new-onset DM (6.9 mmol/L [6.6–7.6] (124.3 mg/dl [118.9-136.9])) and suboptimal controlled DM (7.9 mmol/L [7.4–8.7] (142.3 mg/dl [133.3-156.8])) groups. The overall median TAR was 13.8 % [4.0–26.1]. The optimal controlled DM group had the highest TAR (26.7 % [11.3–49.0]) while the new-onset DM and suboptimal controlled DM group had TARs of 4.6 % [1.2–9.6]and 16.3 % [11.5–23.4], respectively. All perioperative glucose metrics by DM status are presented in Table 3. Notably, the optimal controlled DM group showed considerable inter-individual variability in time in ranges. Individual patient data grouped by DM status are shown in Fig. 4.

**Table 3:** Perioperative glucose metrics for 12 patients.

	All patients (N = 12)	New-onset DM (n = 4)	Suboptimal controlled DM (n = 4)	Optimal controlled DM (n = 4)
Duration of sensor wear, days	13.9 [13.8–13.9]	13.9 [13.9–13.9]	13.9 [12.2–13.9]	13.9 [13.7–13.9]
Mean sensor glucose, mmol/L	7.8 [6.8–8.7]	6.9 [6.6–7.6]	7.9 [7.4–8.7]	8.3 [7.1–9.9]
SD, mmol/L	2.0 [1.3–2.2]	1.3 [1.2–1.7]	2.1 [2.0–2.4]	2.2 [1.5–2.3]
CV, %	23.5 [17.2–28.5]	18.2 [15.5–24.9]	26.8 [25.0–30.1]	22.2 [18.1–26.2]
Time in ranges, %				
<3.9 mmol/L	0.2 [0.0–0.8]	0.0 [0.0–0.2]	0.8 [0.3–1.3]	0.2 [0.0–1.3]
3.9–10.0 mmol/L	85.8 [72.9–95.2]	95.2 [90.4–98.6]	82.9 [76.2–87.3]	72.0 [50.9–87.6]
>10.0 mmol/L	13.8 [4.0–26.1]	4.6 [1.2–9.6]	16.3 [11.5–23.4]	26.7 [11.3–49.0]
>13.9 mmol/L	0.1 [0.0–2.7]	0.03 [0.0–0.5]	2.7 [0.5–5.1]	0.1 [0.0–4.3]
Frequency of episodes, n				
<3.9 mmol/L				
>10.0 mmol/L	0.5 [0.0–3.5]	0.0 [0.0–1.5]	3.5 [1.5–4.5]	0.5 [0.0–3.0]
>13.9 mmol/L	9.0 [6.5–18.0]	7.0 [4.0–9.0]	10.5 [8.5–14.5]	19.0 [10.0–19.0]
	0.5 [0.0–2.0]	0.0 [0.0–0.5]	2.0 [0.5–3.0]	0.5 [0.0–9.0]

All data are depicted as median [IQR].CV = coefficient of variation; DM = diabetes mellitus; min = minutes; SD = standard deviation.

### Patients’ satisfaction regarding CGM sensor wear

**Fig. 5:**
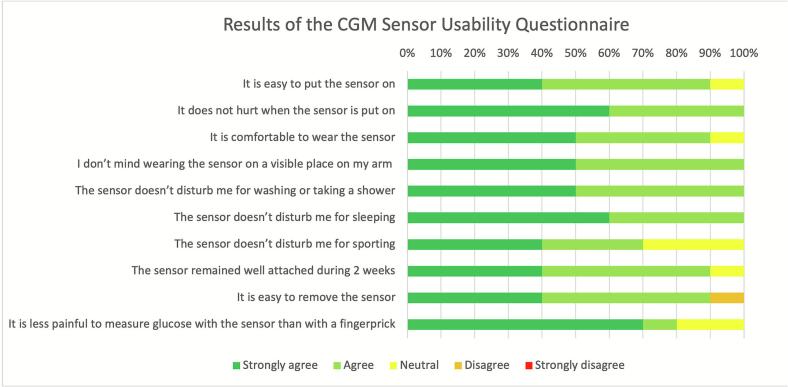
Results of the CGM Sensor Usability Questionnaire. For each statement, responses from 10 participants are displayed as the percentage distribution across all five response categories (1 = strongly agree to 5 = strongly disagree).

No adverse events were reported related to the CGM sensor during the sensor wear period. Of the 15 participants, 12 received the survey invitation. Two patients were not invited as they did not undergo surgery and therefore did not complete the entire CGM period, while one patient died before completing the final CGM period. Of the 12 participants who were invited, 10 completed the questionnaire, yielding a response rate of 83.3 %. Patients reported that sensor application was not painful, they did not mind wearing the sensor on a visible part of the arm, and the sensor did not interfere with washing, showering, or sleeping. All but one participant agreed that the sensor was easy to apply and comfortable to wear. Only one participant reported disagreement with a single item, indicating that the sensor was not easy to remove. The results of the questionnaire are summarized in Fig. 5.

## Discussion

This pilot study shows that nearly all (93 %) patients with (new-onset) DM and pancreatic tumours experienced preoperative hyperglycaemia when monitored using blinded CGM. Patients with suboptimal controlled DM had the highest preoperative TAR, but elevated glucose levels were also common in those with new-onset DM. In the perioperative period, despite institutional glucose control protocols, all patients experienced at least one episode of hyperglycaemia. Interestingly, patients with optimal controlled DM had higher perioperative TAR compared to new-onset and suboptimal controlled DM. Both pre- and perioperative CGM sensor data showed substantial inter-individual differences across persons and groups. Preoperative HbA1c levels declined in most patients, suggesting improved glucose control. Lastly, patients reported high satisfaction with CGM wear, supporting the feasibility and acceptability of sensor wear.

Our findings are consistent with previous research showing that perioperative glucose regulation is frequently suboptimal, even under structured clinical care. In a prospective observational trial using perioperative blinded CGM in patients with type 2 DM undergoing major surgery, 85–90 % of the patients experienced at least one hyperglycaemic episode, with a median TAR of 14–22 %, consistent with our findings [36]. Regarding preoperative hyperglycaemia, another study showed that nearly 80 % of the patients referred for pancreatic surgery have (pre)diabetes [37], indicating a high prevalence of hyperglycaemia in this population as well, which is consisted with our findings. To the best of our knowledge, no previous research is available on pre- and perioperative hyperglycaemia measured with blinded CGM in pancreatic surgery. Our findings show that pre- and perioperative hyperglycaemia have a high prevalence in this high-risk population and that CGM can identify hyperglycaemia, even in patients with seemingly optimal controlled DM based on HbA1c values.

In our study, a patient with new-onset diabetes showed a rapid HbA1c decline from 87 to 25 mmol/mol (10.1 to 4.4 %) following the initiation of insulin therapy. While this suggests improved glycaemic control, abrupt normalization of glucose may worsen complications such as diabetic retinopathy [38]. This highlights the importance of gradual correction of hyperglycaemia and the importance of continuous monitoring to guide glucose-lowering therapy in the preoperative period.

Perioperative hyperglycaemia also persisted despite structured glucose regulation protocols and CGM showed wide variability in TAR. Interestingly, patients with preoperative optimally controlled DM demonstrated the highest TAR. Several factors may contribute to this finding. First, HbA1c correlates poorly with short-term glycaemic fluctuations, which may explain why patients with seemingly optimal controlled DM experienced substantial perioperative hyperglycaemia. Second, perioperative TAR is likely influenced by multiple peri- and postoperative factors, including type and extent of pancreatic resection, nutrition, infection, and the transition from strict ICU glucose protocols to ward care, where strict glucose management protocols are less consistently applied, particularly since not all patients were admitted to the ICU postoperatively [39,40]. In this pilot study we did not have detailed information concerning these parameters. Additionally, the small sample size limits the ability to draw definitive group-level conclusions. Nevertheless, CGM may offer individual benefit by identifying patients who require stricter postoperative glucose regulation.

A key strength of this study is the use of blinded CGM, which provided an unbiased representation of glucose fluctuations. This approach strengthens the validity of our findings and offers a clean insight into the glucose regulation in the pre- and perioperative period. Additionally, patients reported high satisfaction with CGM use, supporting the acceptability and future use of a non-blinded CGM, with the intention to increase time in range and, ultimately, improve outcomes.

Nevertheless, this study has limitations. The small sample size and exploratory design limit generalizability. As noted before, pre- and perioperative CGM data were influenced by multiple factors such as diabetes status, diabetes treatment, type of surgery, ICU admission, postoperative nutrition and complications, making interpretation of the results more difficult and limiting the drawing of group-level conclusions. Next to this, only patients with a high-risk of hyperglycaemia were included, likely resulting in the high incidence of pre- and perioperative hyperglycaemia. Future studies should therefore also include patients without diabetes, to investigate whether this population is also at risk of pre- and perioperative hyperglycaemia. Including this population would help quantify the true burden of pre- and perioperative hyperglycaemia and evaluate whether CGM can identify clinically relevant glycaemic patterns in patients without diabetes. Additionally, our small sample size was not powered for assessing the relation between hyperglycaemia and postoperative outcomes, although this would be clinically informative. Larger prospective studies are needed to determine whether pre- and/or perioperative TAR can predict surgical outcomes.

Despite these limitations, our findings indicate that CGM is a valuable tool for identifying hyperglycaemia in the pre- and perioperative period and that patients with (new-onset) DM scheduled for pancreatic surgery have a high incidence of hyperglycaemia. Therefore, more strict glucose monitoring with CGM in the pre- and perioperative period of this high-risk population is necessary. Evidence from other populations shows that real-time CGM improves glycaemic outcomes within 4–12 weeks, supporting its potential role in preoperative care [41,42]. Outcomes and insights gained in this pilot study merit future studies to assess the effect of real-time CGM on postoperative outcomes in this population.

## Conclusion

Blinded CGM revealed frequent pre- and perioperative hyperglycaemia and high inter-individual variability in TAR among patients with (new-onset) DM undergoing pancreatic surgery. These findings support the need for stricter and more individualized glucose monitoring. The data from this pilot study supports the design of future studies that should focus on the value of real-time CGM during prehabilitation to improve postoperative outcomes.

## CRediT authorship contribution statement

**Heleen Driessens:** Writing – original draft, Methodology, Investigation, Formal analysis, Data curation, Conceptualization. **Josephine A.C. Woldring:** Writing – original draft, Investigation, Formal analysis. **Maarten W. Nijkamp:** Writing – review & editing, Methodology, Conceptualization. **Peter R. van Dijk:** Writing – review & editing, Supervision, Resources, Methodology, Conceptualization. **Joost M. Klaase:** Writing – review & editing, Supervision, Project administration, Methodology, Conceptualization.

## Declaration of competing interest

The authors declare the following financial interests/personal relationships which may be considered as potential competing interests: [Peter R. van Dijk reports a relationship with Abbott that includes: consulting or advisory and funding grants. Peter R. van Dijk reports a relationship with Medtronic that includes: consulting or advisory and funding grants. Peter R. van Dijk reports a relationship with Dexcom that includes: funding grants. If there are other authors, they declare that they have no known competing financial interests or personal relationships that could have appeared to influence the work reported in this paper.].
