# Supplementary material for: Continuous glucose monitoring reveals high prevalence of hyperglycaemia in patients prior to pancreatic surgery: A pilot study
Source: J Clin Transl Endocrinol. 2025 Dec 3;43:100426. doi: 10.1016/j.jcte.2025.100426 (PMC12741282; doi:10.1016/j.jcte.2025.100426)
Supplement: Supplementary Data 1 [file mmc1.docx]

**Supplementary File S1. Glucose Monitor Usability Questionnaire**

We would like to ask you to share your opinion about the new glucose monitor that was applied during this study.

Please circle the number that best reflects your agreement with each statement regarding the new glucose monitor.

| Statement | Strongly Agree (1) | Agree (2) | Neutral (3) | Disagree (4) | Strongly Disagree (5) |
| --- | --- | --- | --- | --- | --- |
| 1. It is easy to put the sensor on | ☐ | ☐ | ☐ | ☐ | ☐ |
| 2. It does not hurt when the sensor is put on | ☐ | ☐ | ☐ | ☐ | ☐ |
| 3. It is comfortable to wear the sensor | ☐ | ☐ | ☐ | ☐ | ☐ |
| 4. I don’t mind wearing the sensor on a visible place on my arm | ☐ | ☐ | ☐ | ☐ | ☐ |
| 5. The sensor doesn’t disturb me for washing or taking a shower | ☐ | ☐ | ☐ | ☐ | ☐ |
| 6. The sensor doesn’t disturb me for sleeping | ☐ | ☐ | ☐ | ☐ | ☐ |
| 7. The sensor doesn’t disturb me for sporting | ☐ | ☐ | ☐ | ☐ | ☐ |
| 8. The sensor remained well attached during 2 weeks | ☐ | ☐ | ☐ | ☐ | ☐ |
| 9. It is easy to remove the sensor | ☐ | ☐ | ☐ | ☐ | ☐ |
| 10. It is less painful to measure glucose with the sensor than with a fingerprick | ☐ | ☐ | ☐ | ☐ | ☐ |
