# Supplementary material for: Continuous glucose monitoring reveals high prevalence of hyperglycaemia in patients prior to pancreatic surgery: A pilot study
Source: J Clin Transl Endocrinol. 2025 Dec 3;43:100426. doi: 10.1016/j.jcte.2025.100426 (PMC12741282; doi:10.1016/j.jcte.2025.100426)
Supplement: Supplementary Data 2 [file mmc2.docx]

Supplementary Table S1. Perioperative characteristics for all patients and by DM status.

|  | All patients (N=15) | New-onset DM (n=5) | Suboptimal controlled DM (n=6) | Optimal controlled DM (n=4) |
| --- | --- | --- | --- | --- |
| Perioperative HbA1c, mmol/mol* | 49.5 [40.5-55.5] | 48.5 [35.5-53.0]* | 56.0 [53.0-72.0]* | 38.0 [37.5-40.5]* |
| Type of surgery  PPPD  PRPD  Whipple  Distal pancreatectomy  No resection | 0  8 (53.5)  2 (13.3)  3 (20.0)  2 (13.3) | 0  4 (80.0)  0  0  1 (20.0) | 0  3 (50.0)  0  2 (33.3)  1 (16.7%) | 0  1 (25.0)  2 (50.0)  1 (25.0)  0 |
| Open procedure** | 12 (92.3) | 5 (100) | 5 (83.3) | 4 (100) |
| Duration of surgery, min** | 449.0 [389.0-578.0] | 417.5 [396.0-583.0] | 449.0 [293.0-472.0] | 543.5 [436.0-603.0] |
| Blood loss, mL** | 500.0 [300.0-800.0] | 500.0 [375.0-550.0] | 350.0 [300.0-550.0] | 1000.0 [500.0-1525.0] |
| Major complications** | 5 (38.5) | 2 (50.0) | 1 (20.0) | 2 (50.0) |
| SSI**  Superficial  Deep | 3 (23.1)  2 (15.4) | 0  1 (25.0) | 1 (20.0)  0 | 2 (50.0)  1 (25.0) |
| DGE** | 3 (23.1) | 1 (25.0) | 0 | 2 (50.0) |
| LOS, days** | 8.0 [6.5-13.5] | 13.0 [7.0-28.0] | 6.0 [5.0-8.0] | 10.5 [7.5-13.5] |
| 30-day readmission** | 2 (15.4) | 0 | 0 | 2 (50.0) |
| 30-day mortality** | 1 (7.7) | 0 | 1 (20.0) | 0 |

Data are depicted as median [IQR] or numbers (%).
DGE = delayed gastric emptying; DM = diabetes mellitus; HbA1c = glycated haemoglobin; LOS = length of hospital stay; min = minutes; PPPD = pylorus-preserving pancreatoduodenectomy; PRPD = pylorus-resecting pancreatoduodenectomy; SSI = surgical site infection
*: For three patients no perioperative HbA1c was determined; one patient in each DM group.
**: Two patients (one in the new-onset DM group and one in the suboptimal controlled DM group) were not operated on; therefore, postoperative characteristics are reported for the 13 patients who underwent surgery.
